# The Role of Trauma and Stressful Life Events among Individuals at Clinical High Risk for Psychosis: A Review

**DOI:** 10.3389/fpsyt.2017.00055

**Published:** 2017-04-20

**Authors:** Danessa Mayo, Sarah Corey, Leah H. Kelly, Seghel Yohannes, Alyssa L. Youngquist, Barbara K. Stuart, Tara A. Niendam, Rachel L. Loewy

**Affiliations:** ^1^Imaging Research Center, Department of Psychiatry and Behavioral Sciences, University of California Davis, Sacramento, CA, USA; ^2^Department of Psychiatry, University of California San Francisco, San Francisco, CA, USA

**Keywords:** clinical high risk, trauma, early psychosis, stressful life events, ultra-high risk, schizophrenia

## Abstract

The experience of childhood trauma (CT) and stressful life events (SLEs) is associated with subsequent development of a variety of mental health conditions, including psychotic illness. Recent research identifying adolescents and young adults at clinical high risk (CHR) for psychosis allows for prospective evaluation of the impact of trauma and adverse life events on psychosis onset and other outcomes, addressing etiological questions that cannot be answered in studies of fully psychotic or non-clinical populations. This article provides a comprehensive review of the current emerging literature on trauma and adverse life events in the CHR population. Up to 80% of CHR youth endorse a lifetime history of childhood traumatic events and victimization (e.g., bullying). Several studies have shown that the experience of CT predicts psychosis onset among CHR individuals, while the literature on the influence of recent SLEs (e.g., death of a loved one) remains inconclusive. Multiple models have been proposed to explain the link between trauma and psychosis, including the stress-vulnerability and stress-sensitivity hypotheses, with emphases on both cognitive processes and neurobiological mechanisms (e.g., the hypothalamic–pituitary–adrenal axis). Despite the preponderance of CHR individuals who endorse either CT or SLEs, no clinical trials have been conducted evaluating interventions for trauma in CHR youth to date. Furthermore, the current process of formal identification and assessment of trauma, SLEs, and their impact on CHR youth is inconsistent in research and clinical practice. Recommendations for improving trauma assessment, treatment, and future research directions in the CHR field are provided.

## Introduction

While a wealth of data has demonstrated indirect associations between childhood trauma (CT) and psychosis in adulthood, the role of CT in the etiology of psychosis and its potential underlying mechanisms are not yet well-understood (1–4). CT is the experience of a highly distressing event or situation during youth that is beyond one’s capacity for coping and/or control (5, 6). Prospective studies of individuals who later develop psychosis provide a unique opportunity to examine potential risk factors, resilience factors, and mechanisms that may link CT and psychosis. Over the past decade, the “clinical high risk” (CHR) paradigm has been used to identify adolescents and young adults at increased imminent risk for developing psychotic disorders. Thus, CHR research makes an important contribution to understanding the potential etiologic role of CT in the development of psychosis. In this paper, we review the emerging literature on trauma and stressful life events (SLEs) in CHR individuals, with a focus on both behavioral and neurobiological studies. This paper also provides a risk model that explains the trauma and psychosis relationship. Further, current and important future directions for assessment, research, and clinical care are highlighted.

### The CHR Syndrome

The CHR syndrome, also termed “ultra high risk” by some research groups, is typically diagnosed using one of two semi-structured interviews—the Structured Interview for Psychosis Risk Syndromes (SIPS) or the Comprehensive Assessment of At-Risk Mental States (CAARMS) (7, 8). The interviews assess a variety of subthreshold psychotic-like experiences (i.e., positive, negative, and disorganized symptoms), general psychopathology (e.g., depression, anxiety), functioning, and family history. Despite slight differences in diagnostic criteria and terminology, both instruments diagnose three possible risk syndromes and have shown high agreement ratings (86%) (9). Other instruments are also used to diagnose individuals at elevated risk for psychosis based on subjective cognitive changes (10, 11). As such, these selected standardized measures have been utilized in international early psychosis (EP) clinics and research programs in an effort to reach diagnostic consensus and validity across sites.

### Psychosis Risk and Outcomes

Outcomes for CHR individuals are heterogeneous: proportion estimates of individuals who transition to full psychosis range from 10–70% due to ascertainment strategy, diagnostic instrument, and follow-up period used (12). The largest individual study using the SIPS demonstrated a conversion rate of 35% to full psychosis by 2.5 years (12, 13). The CHR syndrome confers higher and more immediate risk than heritability estimates of 10% risk among first-degree family members (i.e., parent-offspring; full siblings), although not as high as the 50% rate of psychosis among identical twins (14, 15). Several factors appear to increase the risk for developing psychotic disorders among those with CHR syndromes: poor premorbid functioning, severe positive symptoms (i.e., elevated unusual thought content, increased suspiciousness), increased anhedonia, poor cognition (i.e., impaired verbal learning, decision-making, memory), decline in social and role functioning, substance abuse history, and family history of psychosis (16–19).

It is important to note that approximately 65% of CHR individuals do *not* develop psychosis within the first 3 years after diagnosis of the syndrome. A significant proportion (46%) of non-converters experience remission of their attenuated psychosis (20). Yet, many of those who do not develop psychosis continue to experience psychiatric problems such as mood, anxiety, and substance use disorders (21, 22). By targeting individuals presenting with attenuated psychotic symptoms or other markers indicative of increased psychosis risk, CHR programs seek to identify factors that could be addressed in order to mitigate a variety of negative outcomes and support resilience. To date, a number of potential factors related to outcomes in CHR populations have been identified, including the role of trauma and stress.

## Trauma Experiences in the CHR Population

The experience of CT leads to a cascade of negative effects on typical child and adult development (6, 23). A strong body of literature on the general population of adolescents and young adults (*via* school-wide samples, research and clinical settings, and longitudinal population studies) suggests that CT contributes to poor cognitive, social, medical, and developmental functioning; moreover, CT is a significant risk factor for later development of serious mental illness (SMI), including schizophrenia (24–34). The lifetime prevalence rate of trauma exposure is high among both men (60.7%) and women (51.2%) (35). Individuals ages 14–24 reported exposure to one or more traumatic experiences, such as physical abuse (50%), child abuse, or neglect (13%) (36); approximately 68% of youth by age 16 endorsed at least one trauma experience (37). CT is linked with a variety of adult psychopathology outcomes. Compared to adult participants with no history of CT, those with exposure to four or more traumas were at substantial risk (4- to 12-fold) for developing substance use, depression, and suicidality (38). Thus, several studies on stress and trauma posit that the experience of CT and prolonged early stressors may contribute to the increased risk of future SMI (24–34, 39–41).

To date, only 24 studies, representing 14 distinct samples, report on CT in CHR populations; of these, 11 followed the participants longitudinally to examine CT as a risk factor for developing psychosis (2, 34, 40, 42–62). Sixteen of these studies were included in a recent review and meta-analysis (5), which summarized the existing studies in a series of tables. We refer the reader to this paper by Kraan and colleagues (5) and have summarized more recent publications (8, 34, 45, 49, 51, 52, 55, 59) in a parallel table below (see Table 1).

**Table 1:** **Studies on clinical high risk (CHR) individuals with trauma history and/or stressful life events**.

Reference	Study	Outcome measure	Trauma instrument	CHR instrument	Study design	Participants	Gender (male)	Mean age (range)	Conclusion
Russo et al. (49)	CAMEO United Kingdom; NIHR, United Kingdom	Examine trauma characteristics associated with CHR	THS	CAARMS	2-year follow along	*N* = 60 CHR; *N* = 60 HC	51.7; 43.3%	19.89 (16.41–30.21) 22.6 (16.18–35.57)	Age at study entry, number of traumas, and age at trauma exposure were predictors of CHR group association
Thompson et al. (55)	PACE, Australia	Examine relationship between trauma (specifically sexual trauma) and conversion to psychosis	CTQ	CAARMS	Follow along, length not specified	*N* = 416 CHR [similar sample as Thompson et al. (52)]	Unspecified	Unspecified age and range	Positive correlation between childhood sexual abuse and conversion to psychosis. Relationship unique to sexual trauma.
Kraan et al. (34)	Dutch Prediction of Psychosis Study, Netherlands	Determine the relationship between childhood trauma (CT) and functional/clinical outcome overtime	TADS	SIPS	24-month follow along; follow-up at 9-month, 18-month, and 24-month	*N* = 125 CHR	68.00%	17.7 (unspecified)	Trauma not related to conversion, differential symptom, or functioning overtime. Positive correlations between level of trauma and attenuated positive symptoms, general symptoms, and depression. Trauma negatively correlated with functioning at baseline and follow-up
Üçok et al. (59)	Psychotic Disorders Research Program, Istanbul	Investigate association between CT and CHR cognitive functioning	CTQ	BPRS	Cross-sectional	*N* = 53 CHR	73.60%	21.1 (unspecified)	CHR participants with trauma history had worse attention and working memory. Cognitive flexibility and interference inhibition scores lower than those without a history of CT. No association between trauma and verbal learning/memory. Suggests CT and cognitive deficits may be associated with types of trauma
Yung et al. (61)	PACE, Australia	Examine clinical predictors for poor functional outcomes in CHR patients. Examine a relationship between poor functioning and conversion to psychosis	CTQ	CAARMS	14-year follow along	*N* = 268 CHR	43.20%	Unspecified (15–30)	Childhood maltreatment and psychosis significantly predicted poor functional outcome. No association between positive symptoms and follow-up functioning. Cross-sectional relationship found between long-term poor functioning and negative symptoms at follow-up in both converters and non-converters
Kline et al. (45)	Strive for Wellness, Maryland	Examine relationship between trauma and early psychosis and psychosis risk symptoms in youth	KSADS-PL	SIPS	Cross-sectional	*N* = 60 CHR/EP; *N* = 65 LR	49.00%	15.88 (unspecified)	Trauma history related to positive symptoms in both groups. LR group reported heightened suspiciousness with a history of exposure to violence. CHR/EP group reported heightened levels of suspiciousness regardless of type of violence exposure
Stowkowy et al. (51)	NAPLS-2, North America	Determine whether trauma and discrimination are predictors of conversion to psychosis	Childhood Trauma and Abuse Scale; Adapted self-report measure used for perceived discrimination	SIPS	Cross-sectional	*N* = 764 CHR; *N* = 280 HC	55.30%	18.5 (unspecified) 19.7 (unspecified)	CHR group reported higher levels of trauma, perceived discrimination, and bullying than HC. Discrimination was a significant predictor of conversion. Discrimination correlated with ethnic minority groups
Thompson et al. (52)	PACE, Australia	Examine if certain factors mediate the relationship between sexual trauma and psychosis	CTQ	CAARMS	Follow along, 2.4–14.9 years later	*N* = 416 CHR	Unspecified	Unspecified (15–30)	Anxiety, dissociation, mood instability and mania symptoms did not mediate the relationship between sexual trauma and psychosis

*Trauma measures: THS, Trauma History Screen; TADS, Trauma and Distress Scale; CTQ, Childhood Trauma Questionnaire; PACE, Personal Assessment and Crisis Evaluation*.

*Psychiatric assessments: MINI, Mini International Neuropsychiatric Interview Version 6.0.0; PANSS, Positive and Negative Symptom Scale; SIPS, Structured Interview for Psychosis Risk Syndromes; KSADS-PL, Kiddie Schedule for Affective Disorders and Schizophrenia-Present and Lifetime; BPRS, Brief Psychiatric Rating Scale-expanded; SANS, Scale for the Assessment of Negative Symptoms; SAPS, Scale for the Assessment of Positive Symptoms; CAARMS, Comprehensive Assessment of At-Risk Mental State*.

*Studies: NAPLS-2, North American Prodrome Longitudinal Study-2; NIHR, National Institute for Health Research Mental States; CHR, clinical high risk; EP, early psychosis; LR, low risk; HC, healthy control*.

The meta-analysis concluded that CT is a largely prevalent experience among the CHR population (86.8%) compared to healthy controls (HC) (5). Such alarmingly high rates of CT endorsed by the CHR population is comparable to the prevalence rate among individuals with schizophrenia (85%) (5). We expand upon the meta-analysis by reviewing different types of trauma in the CHR syndrome based on all current available information below.

### Associated Findings on Trauma in the CHR Population

Clinical high risk individuals may be at risk for experiencing various forms of traumatic experiences that are common within the general population. The meta-analysis by Kraan and colleagues (5) reported a mean prevalence rate of 86.8% CT in CHR studies (2, 40, 42, 51, 56). The range of rates (35.9–70%) may be partially explained by the type of trauma being examined and the gender sample distribution (e.g., sexual abuse) (40, 53) and type of assessment used (e.g., medical records review, semi-structured interview, self-report), with self-report measures associated with higher rates of trauma disclosure [e.g., Childhood Trauma Questionnaire (CTQ), Early Trauma Inventory] (40–91%) (40, 42, 44, 55, 59). Some measures assess narrowly defined trauma, while others are more broad, including major life events. Eight of the studies were based on small samples of less than 100 participants, with some as small as 25 subjects (40, 44, 46, 50, 53, 56, 58, 62). Smaller samples are more easily biased by sampling differences and thus contribute to heterogeneity of results and lack of reproducibility (63). The largest sample reported 46.2% of CHR individuals with CT (*n* = 764), a majority of those who endorsed severe to extreme rates of trauma on the brief CTQ (51, 52, 55). As a whole, these studies highlight the preponderance of CT experiences among the CHR population and the importance of such information for clinical consideration.

Four studies formally compared CT rates in CHR samples with HC populations matched on demographic variables (i.e., age, gender, socioeconomic status) and found higher rates of abuse among CHR groups (2, 50, 58). Research suggests that CHR individuals may be at greater risk for physical trauma than the general population (17%) (64). In a small study of 30 CHR participants, 83% reported a physical abuse history (56). A study on a CHR sample reported more violent (71.7%) and non-violent events (53.3%) than the low-risk group (48.4% violent; 33.9% non-violent events) (45). Physical trauma is also associated with poorer cognitive functioning, which is a significant concern for CHR individuals, as poor premorbid cognitive functioning may add to their psychopathology risk (59, 61).

Individuals with a sexual abuse history are at higher risk for developing mood and anxiety disorders, substance abuse, posttraumatic stress disorder (PTSD), eating disorders, suicidal behaviors, and psychosis (65–69). Across studies, approximately 22–31.1% of CHR individuals endorsed a sexual trauma history (34, 40, 44, 49, 52, 53, 56) compared to the lifetime prevalence rate in the general population (15–25%) (70). Similarly, a study of 92 CHR individuals with a sexual abuse history endorsed higher rates of positive symptoms of a sexual nature (e.g., feelings of being watched while bathing, hearing voices say sexual statements) than HC (53). This may indicate that previous experiences of sexual trauma contribute in part to the nature of CHR individuals’ emerging positive psychotic symptoms. CHR youth showed even higher rates of emotional abuse (41.5–75%) and emotional neglect (59–100%) (56, 58, 59) compared to HC (33%) (58). Furthermore, emotional abuse and neglect among CHR individuals have also been associated with greater Schneiderian first-rank symptoms, more elevated Schneiderian total score, and depression severity level (2).

Bullying victimization is becoming increasingly recognized as an important form of adverse childhood experience (24). Bullying has been associated with a variety of poor outcomes, ranging from poor self-esteem, depression, suicidality, aggression, and psychosis being the most serious (71). CHR youth endorsed a lifetime history of physical and psychological bullying (30 and 60%, respectively) that was much higher than HC (14 and 36%, respectively) (42). Bullying history among CHR youth was significantly associated with poorer social functioning (42) and was more likely to persist into adult psychiatric disorders (71). As such, it is imperative that more research is conducted to examine the relationship between childhood bullying and psychosis symptoms.

Trauma is often experienced as a result of developing psychosis, due to the experience of frightening psychotic symptoms or hospitalizations, especially involuntary treatment. Prevalence of psychosis-associated trauma symptoms among individuals with full psychotic disorders varies from 11–67% (72, 73). They may be associated with factors such as trauma history prior to inpatient hospitalization (e.g., physical or sexual abuse) and other psychological factors (e.g., negative event appraisals, poor coping skills) (74, 75). However, some studies did not find any associations with psychosis-related trauma symptoms and the number of negative experiences from inpatient psychiatric hospitalizations (73, 76). It may be important to ascertain whether other patient-level factors, such as the level of distress attributed to the inpatient hospitalization, legal status, and involuntary hospitalization may be associated with psychosis-related trauma symptoms (73, 75, 76). To date, there are no specific studies that focus on trauma symptoms associated with psychiatric hospitalization among CHR individuals. This is an important research area in need of further exploration and highlights the usefulness of examining the impact of CT in the CHR population, prior to the potentially traumatizing effects of involuntary hospitalization that can accompany the onset of a full psychotic disorder.

Only a few published studies have explored the demographic characteristics of trauma in CHR individuals. A study on gender differences showed that stress-sensitivity scores among CHR females (but not males) mediated the association between trauma and attenuated positive psychotic symptoms, which suggests that females cope with trauma differently and tend to internalize their experiences (77). While ethnic sample variability (i.e., majority Caucasian women) was a possible research limitation, this is consistent with the common finding that females with psychosis are more likely to endorse a trauma history (e.g., sexual abuse) than males (42, 53) and are more likely to have an affective disorder associated with their diagnosis (78). Similarly, limited studies regarding ethnicity (e.g., perceived discrimination, social adversity) and its relationship with trauma in CHR groups currently exist (51). The current collective studies on CHR groups with trauma experiences suggest the importance of continued research into its associated influence on psychosis risk.

### Trauma and CHR Conversion to Psychosis

Trauma has been repeatedly found to predict transition to psychosis in CHR samples. Sexual abuse is the most common form of CT associated with later psychosis conversion, followed by physical abuse (3, 40, 53, 55, 60). Moreover, emotional abuse and physical neglect have been identified as potential risk factors for psychosis conversion (58). Similar to findings on sexual abuse history, the increased severity and duration of individuals’ bullying history has been linked to the emergence of psychosis symptoms (71). While elevated rates of trauma history were found among CHR individuals in the NAPLS sample and trauma history was a significant predictor of psychosis conversion in the univariate and multivariate analyses, it was not a statistically significant predictor after controlling for prodromal symptom severity, social functioning decline, verbal learning, and memory (16). Thus, the power of trauma to predict conversion must be examined in the context of other predictors in order to determine its relative impact and possible relationship to other predictive factors.

## SLEs in the CHR Population

It is not yet clear whether the impact of trauma on individuals with CHR is specific to narrowly defined traumatic events or also includes the cumulative effects of adverse or SLEs that have also been linked with adult psychopathology, including psychosis risk (79, 80). In fact, many studies that purport to measure traumatic events include less severe SLEs, which consist of dangerous or life-changing experiences that have occurred for an individual (5) and may cause disruption in the typical developmental trajectory of youth through adulthood. Exposure to SLEs are associated with increased risk for depressed mood, anxiety, eating disorders, suicidality, substance use, and psychosis symptoms in later adolescence (29, 81–85). Current findings on the SLE–psychosis risk relationship are inconclusive; some cited a positive relationship (39, 51, 86, 87) while others did not (43, 47, 48, 57). Kraan and colleagues (5) indicated that recent SLEs were less commonly endorsed by CHR youth than HC, which may be due to increasing negative symptoms of psychosis (e.g., increased avolition, social withdrawal) that limit activities. Increased research efforts are underway to improve current understanding of the relationship between SLEs and psychosis.

## Comorbid Disorders and Differential Diagnosis in the CHR Population

The specificity of the relationship between trauma and CHR symptoms is muddied by the high level of comorbidity in this population (88–90). Around 73% of CHR individuals have at least one other Axis I disorder (89). Long-term studies show persistence of comorbid disorders such as mood (15–38%), anxiety (5–16%), substance use (11%), personality disorders (2.7%), and other diagnoses (43–52%) (91, 92). At a 6-year follow-up, approximately 56.8% of CHR patients endorsed at least one comorbid disorder and 61.5% of them reported continued comorbidity from baseline (91). Only a small group of CHR individuals (7–16%) reported no comorbid diagnoses at baseline or follow-up (91, 92). Clearly, comorbidity is the rule and not the exception when dealing with EP symptomatology. Since up to 65% of CHR individuals do *not* go on to develop psychotic disorders within 3 years after initial CHR diagnosis, such subthreshold psychotic symptoms experienced may have responded to treatment, resolved over time, or may be better explained by another psychiatric diagnosis. In a strict sense, these non-converters may be considered “false positive” diagnoses regarding a pre-psychotic phase of illness. Thus, the CHR syndrome may best be understood as a mixture of individuals identified prior to the onset of psychosis, along with adolescents/young adults who experience subthreshold positive symptoms in the context of a primary mood, anxiety or PTSD. Any relationships to trauma must be understood in this context, given the extensive literature that links trauma to later mood and anxiety disorders. In this section, we examine the various comorbid disorders within the CHR and other psychotic disorders and their symptom interaction with CT.

### Mood/Anxiety Disorders

Clinical high risk individuals with CT showed high comorbid mood (40–45%) and anxiety disorders (15.3%) (5, 42, 93), as did CHR individuals with SLEs [i.e., major depressive disorder (13%), bipolar disorder (8.7%), dysthymic disorder (4.3%), social phobia (17.4%), generalized anxiety disorder (8.7%), panic disorder (4.3%)] (57). However, a study on CHR samples was unable to show a relationship between CT and mood/anxiety disorders, possibly due to low sample size (*n* = 30) (56). Available studies on CHR and first-episode psychosis (FEP) individuals with CT show that both groups had higher rates of suicidal attempts, elevated rates of psychiatric hospitalization, and poorer clinical functioning (3, 59). Studies on individuals with schizophrenia suggest that CT is associated with increased severity of depression and anxiety disorders (94, 95). The small number of current findings related to comorbid mood and anxiety disorders among CHR groups with CT warrants additional research in this area to untangle whether trauma is specifically related to positive psychotic symptoms.

### Substance Use

Despite high rates of comorbid substance use in CHR populations, particularly tobacco (34.4%), alcohol (17–44%), and cannabis (3–54%) (96), there is currently limited research specifically focused on CHR individuals with trauma and substance use. However, there are well-documented links between trauma and substance use in the general population (97) and the role of substance use in triggering psychotic episodes (98, 99). While there is minimal support as of yet for the direct relationship between substance use and conversion to psychosis in the CHR group, there is stronger evidence for the relationship between substance use and increased severity of subthreshold psychosis symptoms among CHR individuals (96). School-aged youth showed an interaction effect between CT and cannabis use that accounted for 83% of their reported psychosis symptoms (100). Better understanding potential interactions between trauma and substance use as risk factors for psychosis is a critical need in the literature, as well as highly relevant to designing interventions for this population.

### Posttraumatic Stress Disorder

Most critical to comorbidity issues in our review of trauma and the CHR syndrome is the presence of PTSD in this population. A multisite CHR study reported a significantly higher prevalence rate of current (2.6%) and lifetime (4.1%) formal PTSD diagnosis than in HCs (101). Specifically, CHR youth with a CT history demonstrates PTSD rates of 15.2% (3). Similar to mood and other anxiety disorders, comorbid PTSD diagnoses with FEP individuals who have a trauma history are associated with longer treatment duration and more intensive treatment to address all presenting symptoms (3). A meta-analysis indicated that individuals who suffer from comorbid psychosis and PTSD endorse symptoms of faulty cognitive appraisals, feelings of helplessness, and lack of control (75). These are, of course, important targets for treatment with individuals who have comorbid PTSD and psychosis, and may be relevant for CHR treatment.

### Differential Diagnosis of PTSD and CHR Status

A common referral question posed by clinicians seeking evaluation for a consumer asks— “Is it trauma or EP?” Indeed, the symptoms associated with PTSD can create diagnostic uncertainty. There are a number of similarities between the symptoms of PTSD and psychosis (102). Hallucinations in psychosis are analogous to the experience of flashbacks and intrusive images and bodily sensations associated with PTSD as they both present in visual, auditory, or tactile modalities and are usually experienced as distressing and unbidden. Suspiciousness in psychosis resembles the hypervigilance in PTSD and avoidance behaviors, which are a hallmark of PTSD and can be similar to safety-seeking behaviors or negative symptoms in psychosis. Hallucinations in an adolescent sample were found to be highly prevalent in both PTSD and psychotic disorders, and the hallucinations of psychosis and PTSD could not be differentiated in terms of content, modality, location, or form (103, 104).

It may also pose additional difficulties when evaluating individuals with more severe symptoms. Data from the U.S. National Comorbidity Survey Part II indicated that all of the positive psychotic symptoms examined in the sample were more likely to be endorsed by respondents who met diagnostic criteria for PTSD than those without PTSD (105). A dose–response relationship was also found, such that with more PTSD symptoms endorsed, the higher likelihood for experiencing both symptoms of paranoia and hallucinations. Among the psychotic symptoms, auditory hallucinations had the greatest odds ratios with lifetime PTSD diagnosis. Several studies on non-clinical, community samples suggest that CT is a strong risk factor for visual, auditory, and tactile hallucinations (106, 107). Data from the National Comorbidity Survey indicated that a history of childhood rape was significantly associated with auditory hallucinations in a non-clinical adult (Age M = 32 years, SD = 10.59) sample (107).

Despite the substantial similarities, one study of adolescents suggests that command hallucinations and derogatory themes were more common in PTSD and were associated with higher emotional distress, self-injury, and suicidal ideation, compared to hallucinations in schizophrenia (103). Furthermore, the presence of PTSD in children and adolescents has been noted to confer a substantial likelihood of disturbances of reality testing. Maltreated and traumatized children with a PTSD diagnosis are more likely than children with a history of trauma with no PTSD diagnosis to also meet criteria for a brief psychotic episode or unspecified psychotic disorder with symptoms analogous to the CHR syndrome (108). However, children with PTSD rarely exhibited full-blown delusions or illogicality. Thus, perceptual disturbances and suspiciousness may be present both in the CHR syndrome and in PTSD while other types of delusional thinking, cognitive disorganization, and negative symptoms (differentiated from mood disturbance or avoidant behaviors) may be more specific to psychotic disorders.

## Mechanisms of Trauma and Stress in the CHR Population

Multiple models have been cited to explain the link between trauma and later psychosis, including the stress-vulnerability and stress-sensitivity hypotheses, with emphases on both cognitive processes and neurobiological mechanisms (e.g., the hypothalamic–pituitary–adrenal axis). Consistent with a gene–environment interaction model, trauma history appears to contribute to psychosis in adulthood somewhat independent of genetics (109). A recent gene–environment interaction study supports the idea that genes associated with schizophrenia lead to changes in not only dopamine but also serotonin signaling pathways in the brain, thus suggesting an “affective pathway” to psychosis (110). Below, we address models that have been referenced in previous works to explain the potential interplay between trauma, stress, and psychosis. Following the discussion of currently identified relationship models of trauma and psychosis, we propose our own comprehensive model that conceptualizes a cyclical relationship between trauma and psychosis risk.

### Stress-Vulnerability Model

**Figure 1:**
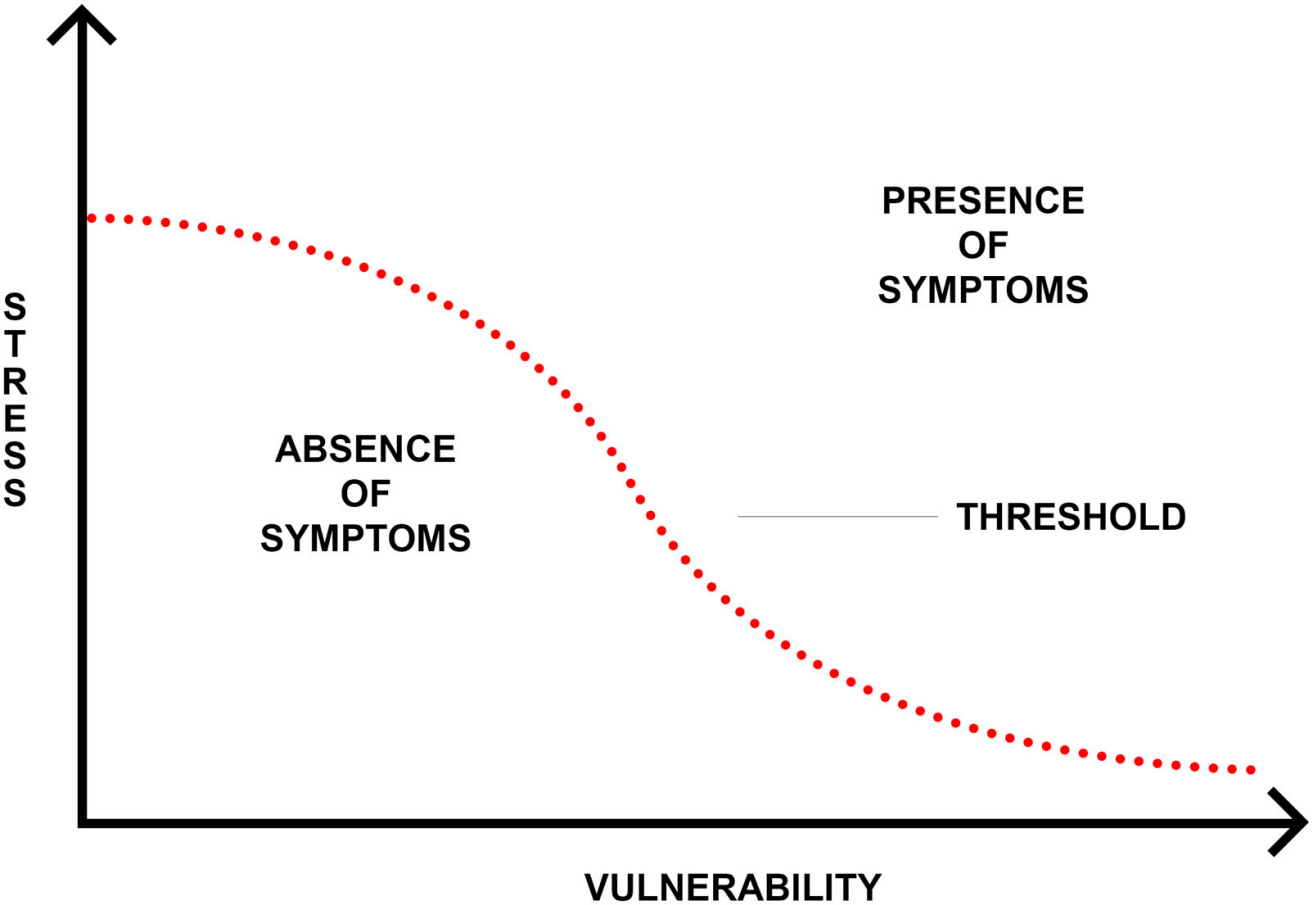
**Stress-vulnerability model (111)**.

**Figure 2:**
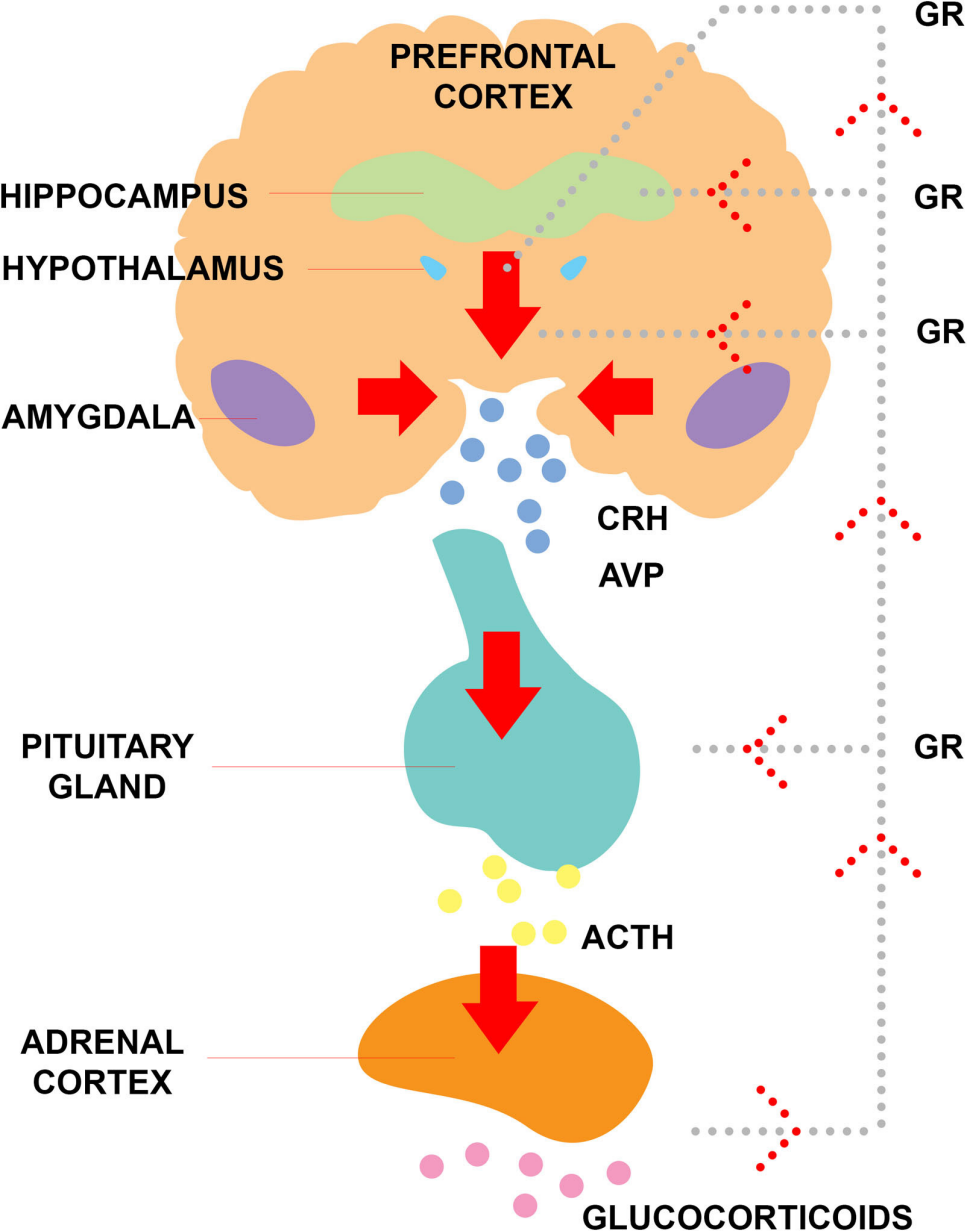
**Functioning of the hypothalamic–pituitary–adrenal axis**. Abbreviations: GR, glucocorticoid receptor; ACTH, adrenocorticotropic hormone; CRH, corticotropin-releasing hormone; AVP, arginine vasopressin.

In an effort to understand the mechanism through which trauma and stress may lead to psychosis, Zubin and Spring (111) proposed the stress-vulnerability model. This model posits that individuals possess a genetic or biological vulnerability to psychosis that can withstand a certain amount of stressors due to genes and other biological risk factors. However, once the stress threshold is surpassed, psychosis may be at higher risk of development (see Figure 1) (111). From this perspective, the experience of trauma increases one’s experienced stress and, therefore, leaves them at greater susceptibility to experiencing psychopathology. One way this has been examined biologically is through research on the functioning of the hypothalamic–pituitary–adrenocortical (HPA) axis (see Figure 2), one of the primary stress response systems in the human body.

### Stress-Sensitization Model

The impact of stress on psychopathology has also been discussed in the context of stress sensitization (112). The origins of this concept come from animal models that indicated individual differences in stress-sensitivity and -reactivity due to interactions between genes and environments. Similar to the stress-vulnerability model, the stress-sensitization theory hypothesizes that for a person to experience their first psychiatric illness, they may have a biological vulnerability, and then need to experience a major stressor. After the initial emergence of psychopathology, vulnerability increases, requiring less stress for the person to develop recurrent or more severe psychiatric issues (113). Thus, experience of CT may render an individual more susceptible to psychosis triggered by later stressors. Walker and Diforio (114) describe the connection of behavioral and biological stressors in psychosis and how dysregulation of stress response in both of these capacities overtime can create even greater disturbances in the HPA axis, thereby creating even more damaging effects on one’s functioning. Support for the concept of a dysregulated response to stress following trauma can be seen in other mental health conditions. In a study of 18,713 individuals without a psychiatric diagnosis prior to the September 11 attack in the US, those who reported a CT history and were either directly or indirectly affected by the attack were at significantly greater risk of experiencing an internalizing disorder and were more vulnerable to elevated stress in comparison to those that did not report CT (115). Within the CHR population, the stress-sensitization model has never been formally tested; that is, no study has examined the possibility of an interaction between CT or SLEs and raising the risk for psychosis onset. A very large sample would be required to test an interaction model to predict conversion to psychosis, which one would only expect for up to a third of CHR individuals. However, partial behavioral evidence for stress sensitization was reported from the NAPLS study, in which CHR individuals who converted to psychosis not only reported more SLEs but also experienced higher levels of self-reported stress than CHR participants whose symptoms remitted. Additionally, those who rated more SLE also indicated higher stress from daily hassles (116). However, they did not examine any relationship of CT to SLE response. We have begun to investigate this possible interaction in our own work as described below.

### Dysregulated Stress Response

Cortisol is a biological stress marker, the final product of activation of the HPA axis in response to stress and can be obtained through plasma, saliva, and urine (117). Cortisol has a standard diurnal rhythm that can be assessed when samples are collected throughout the day (118). In addition to quantifying its daily cycle, stress reactivity can be measured when cortisol is measured in saliva samples that have been collected when a person is undergoing a stressful task (119). Therefore, dysregulation of the HPA axis can be evaluated globally to address the underlying vulnerability to stress, and locally, sensitivity can be evaluated in response to a specific stress task. Findings within the schizophrenia population indicate elevated cortisol (a physiologic measure of stress responsivity) in many individuals with psychosis (120, 121), with some variable results indicating both hyper- and hypo-function of the HPA axis (122). Some of the heterogeneity in results could be due to the presence of antipsychotic medication, which has shown to decrease cortisol levels (122, 123) or potentially to the experience of trauma.

### Trauma and the HPA Axis

There is currently some evidence for the impact of trauma on the development of altered stress responses and psychopathology. Indeed, research has shown blunted cortisol secretion in patients with PTSD (124), and in women with a history of sexual trauma (125), as well as for patients with schizophrenia who reported CT. In a study of 14 individuals who met diagnostic criteria for DSM-IV schizophrenia, those who reported moderate to severe CT experiences had lower diurnal cortisol secretion, especially within the first hour of the waking, whereas those without the experience of CT exhibited higher levels of cortisol throughout the day (126). Following the same pattern, Phassouliotis and colleagues (127) found lower basal cortisol levels, in a sample of first-episode patients who reported significantly higher rates of CT than HC. However, due to the small sample size, within-group comparisons of first-episode patients with and without CT could not be explored. Within the CHR population, this theory has had a lack of attention, but one study has supported the idea. Although trauma was not explicitly assessed, decreased cortisol secretion as a response to Trier Social Stress Test administration was found in a small sample of CHR individuals who also reported higher levels of chronic stress compared to HCs (119). Further research evaluating CT history explicitly within the CHR population is necessary to understand its impact on cortisol secretion.

Evidence suggests that psychosocial stress activates the HPA axis and, in turn, the dopamine and serotonin systems, where exaggerated effects have been observed in individuals who experienced childhood adversity (110, 128). Neuroimaging studies (i.e., magnetic resonance imaging; functional magnetic resonance imaging; positron emission tomography; diffusion tensor imaging; multimodal) have revealed that in those CHR individuals who converted to psychosis, functional changes in striatal dopamine synthesis and release were observed (129–131). In addition, Oswald and colleagues (132) found that perceived stress partially mediated the association between childhood adversity and ventral striatal dopamine responses. A full exploration of the potential neurobiological mechanisms linking trauma and psychosis are beyond the scope of this paper, but we provide these examples of one possible relationship and highlight the need for further research in this area.

Overall, a majority of studies has demonstrated abnormalities in cortisol secretion in CHR samples compared to HC (133). In a large sample of 256 CHR patients and 141 HC, the CHR group exhibited significantly higher mean diurnal salivary cortisol levels (134). Participants who converted to full psychosis in the CHR group had higher mean daily cortisol levels than those who remitted. However, the specificity of this result to psychosis is unclear, as well as whether it is a cause or consequence of attenuated psychosis. It may be related to the high rates of mood and anxiety disorders in this group rather than be central to psychosis, specifically. Its relative contribution to psychotic transition in the context of other significant risk factors is also not yet fully understood. Additional research that follows HPA axis functioning overtime and relates it to symptom expression and other biomarkers, such as genetics and neuroimaging, are critical to understanding the role it may (or may not) play in psychosis risk. A dysregulated stress response with altered cortisol secretion may be evidence of a subgroup of CHR individuals who experience an affective/stress pathway to psychosis, and thus moderating the stress response at a biological or behavioral level could be an important target for intervention in those with a demonstrated dysregulated response.

### Cognitive Mechanisms

Several cognitive mechanisms may explain the associated link between trauma and psychotic disorders. For instance, it has been suggested that early adversity may lead to the formation of negative schemas of the self, others, and the surrounding environment (135). Such negative views may eventually contribute to greater external locus of control (54) and increased symptoms of suspicious or paranoia (136). CT may be associated with faulty responses to environmental stimuli, such as informational processing bias for negative or irrelevant stimuli (137, 138). Such focus on irrelevant or what may appear to be threatening stimuli has been thought to lead to reasoning bias (e.g., jumping into conclusions) (136, 139) and paranoid thinking (140). For a more detailed overview of cognitive and neurobiological mechanisms involving trauma and psychosis, we refer the reader to a recent review by Gibson and colleagues (141).

### The Cycle of Trauma, Psychosis, and Future Risk of Trauma

To comprehend the impact of CT and SLE on the development of psychosis, the synthesized findings point to the cyclical nature of trauma, psychosis risk, and increased vulnerability for future traumatic experiences. However, given the current weak findings associating SLE with CHR transition to psychosis, our conceptualized model focuses primarily on CT. Individuals exposed to CT are at elevated risk for abnormal childhood development in terms of neurocognitive, social, and emotional functioning. Depending on the form and severity of CT, some may have a more negative impact on learning and development. For instance, exposure to physical abuse or witness of domestic violence can create globally negative views of the self, others, and the world (142). Maladaptive behaviors (e.g., non-suicidal self-injury, suicidal behaviors, aggression), poor coping skills, and impaired emotional regulation may also arise out of CT experiences, increasing one’s risk for developing mood and anxiety disorders (143). These same behaviors can result in poorer role and social functioning overtime (e.g., bullying, increased peer isolation), thus decreasing protective factors, such as social support and adaptive problem-solving skills. Several studies have also highlighted that the sole experience of trauma does not predict poor clinical functioning and CHR status (34, 52). Instead, as explained by the stress-sensitivity model, one’s trauma history creates an initial level of elevated vulnerability for later psychopathology, such as SMI. Other risk factors (e.g., genetic, environmental) may add or interact with trauma to confer increased risk for psychosis. While the current evidence does not support SLE as a trigger for psychosis onset, they do lead to increased levels of depression in both episodes and severity (143). Severe mood disorders that are characterized with psychotic features are often more difficult to treat (144, 145). Thus, the dose–response relationship suggests that with increased CT experiences, the risk for later psychosis becomes greater.

Unfortunately, the negative impact of CT does not end at the onset of psychotic illness. For CHR individuals who endorsed CT, the emergence of psychosis creates a string of increased vulnerability for future traumatic experiences. CHR individuals with CT who go on to full psychosis conversion tend to have poor long-term functioning outcomes (61). In general, increased psychosis risk is associated with a decline in global functioning (e.g., social, role), emergence of comorbid disorders (e.g., depressed mood and anxiety disorders, PTSD, substance use), poor treatment engagement, and increased maladaptive coping skills and behaviors (16–19). The influence of CT further adds to the complexity of their symptom presentation and severity. As a result, the cascade of abnormal development and increased psychopathology leads to the resurgence of future vulnerability to other trauma.

The trauma–psychosis risk relationship postulates that following an initial traumatic experience, an individual experiences an abrupt change in their normal developmental course, is weakened in various areas of functioning, and therefore, is made more vulnerable moving forward in development. Based on the interaction between an individual’s genetic foundation and their interaction with environmental stressors, including increased stress sensitization, the risk of psychosis conversion escalates. With the onset of full psychosis, individuals are further weakened in their ability to adaptively respond to stressful situations and adverse events moving forward, leading to increased risk of experiencing additional future trauma/SLEs. Nevertheless, the findings which highlight no associations between trauma and transition to full psychosis offer hope that there may be strong protective factors that can be bolstered during treatment of early subthreshold psychotic symptoms or that there may be additional risk factors that can help identify a subgroup of CHR individuals at particular risk for worsening psychosis related to CT.

## Trauma Assessment in the CHR Syndrome

To better understand the role of trauma in the CHR syndrome, current methods of trauma assessment must be harmonized. Although they are used regularly with individuals with psychosis or the CHR syndrome, no existing trauma or SLE measures have been developed or validated specifically with these populations. The National Child Trauma Stress Network (146) and the American Academy of Child and Adolescent Psychiatry (147) provide guidelines on the appropriate assessment and treatment of children and adolescent who may have experienced trauma. These guidelines stress the need to briefly screen all children in a given setting for the experience of traumatic events and, in the presence of a positive screen, to follow up with a more detailed assessment in order to appropriately guide treatment planning.

Trauma experiences and associated clinical consequences can be identified through a variety of methods. Brief self-report screening questionnaires retrospectively assess for the occurrence and reaction to a variety of traumatic events. Brief screening measures for assessing only exposure to traumatic events include the Brief Trauma Questionnaire (148) and the CTQ (149). Measures examining both the experience of trauma *and* its psychological impact (e.g., assesses symptoms and distress) include the Trauma Symptom Checklist for Children (150) and the Child Posttraumatic Symptom Scale (151). The UCLA PTSD Reaction Index for DSM-IV (152) is an example of a combined measure that starts with a brief questionnaire to assess for a history of traumatic events, which is then followed by a semi-structured interview to determine distress and impact of those events to support a diagnosis of PTSD. Some measures also include a collateral informant report, such as the Trauma Symptom Checklist for Young Children (153). Finally, semi-structured diagnostic interviews such as the Structured Clinical Interview for DSM-IV Axis-I Disorders (SCID-I) (154) and the Kiddie Schedule for Affective Disorders and Schizophrenia (155) include sections to assess for a history of traumatic events and their clinical sequelae. Measures most commonly used in CHR research studies are the CTQ, Trauma History Screen, and the CT and Abuse Scale. For a comprehensive list of trauma experience and symptom measures, please refer to the National Child Traumatic Stress Network and the American Academy of Child and Adolescent Psychiatry resources (146, 147).

While various measures of traumatic experiences and SLEs are available, there may be concerns about using them with CHR individuals. For instance, if there are concerns about early trauma experiences being distorted by the delusional thinking (e.g., suspiciousness), collateral reports from family, previous treatment providers, and school staff can clarify the validity of the individual’s reported experience. Conversely, it may be difficult to determine if certain attenuated symptoms (e.g., suspiciousness) are associated with reality-based experiences of victimization and better accounted for by a trauma reaction than a psychotic-spectrum symptom. However, a previous study that has examined the reliability of reported CT experiences by individuals with psychotic disorders found that they were retrospectively accurate and stable over time irrespective of current psychopathology (156). Patients may tend to underreport CT while in treatment for psychosis and could be more forthcoming when experiencing more severe psychotic symptoms. Nevertheless, studies have tackled this issue by enforcing other measures for additional precaution. A UK study on a national sample of (*n* = 2,172) 12-year-old twin children showed that research protocol can easily be structured to determine credibility of children’s reporting in clinical interviews, such as enforcing a rating system that codes from 0 (i.e., not a symptom), 1 (i.e., a likely symptom), to 2 (i.e., definite symptom); and enlisting the clinical judgment of various professionals who are familiar with the CHR group/symptoms within the psychosis spectrum or whose area of specialization is with youth (41). Additionally, clinicians may worry that discussing trauma as part of an evaluation can trigger worsening of psychotic symptoms, which may lead to avoidance of appropriate assessment. Contrary to this belief, research suggests that appropriate and sensitive evaluation of trauma does not increase subjective distress (157, 158).

One significant challenge of concordant trauma and SLE assessment in this population is that the age of individuals often ranges from 12–30, and instruments are often designed for either children, adolescents, or adults. The types of trauma and SLEs experienced by these different age groups vary considerably, with school and family-related stressors (e.g., divorce of parents) relevant for children and work and romantic relationship-related stressors (e.g., one’s own divorce) more relevant for young adults. The measurement of trauma and stressors overtime in the same individual creates challenges in the consistent use of instruments. While a variety of appropriate measures exists to aide in the identification of trauma, there is currently no standardized trauma evaluation protocol for CHR groups as part of research or clinical practice. This lack of detailed information related to trauma or SLE that can be compared across clinics and research precludes our ability to effectively target treatment or elucidate relationships in research.

### CHR and Trauma Interventions

**Figure 3:**
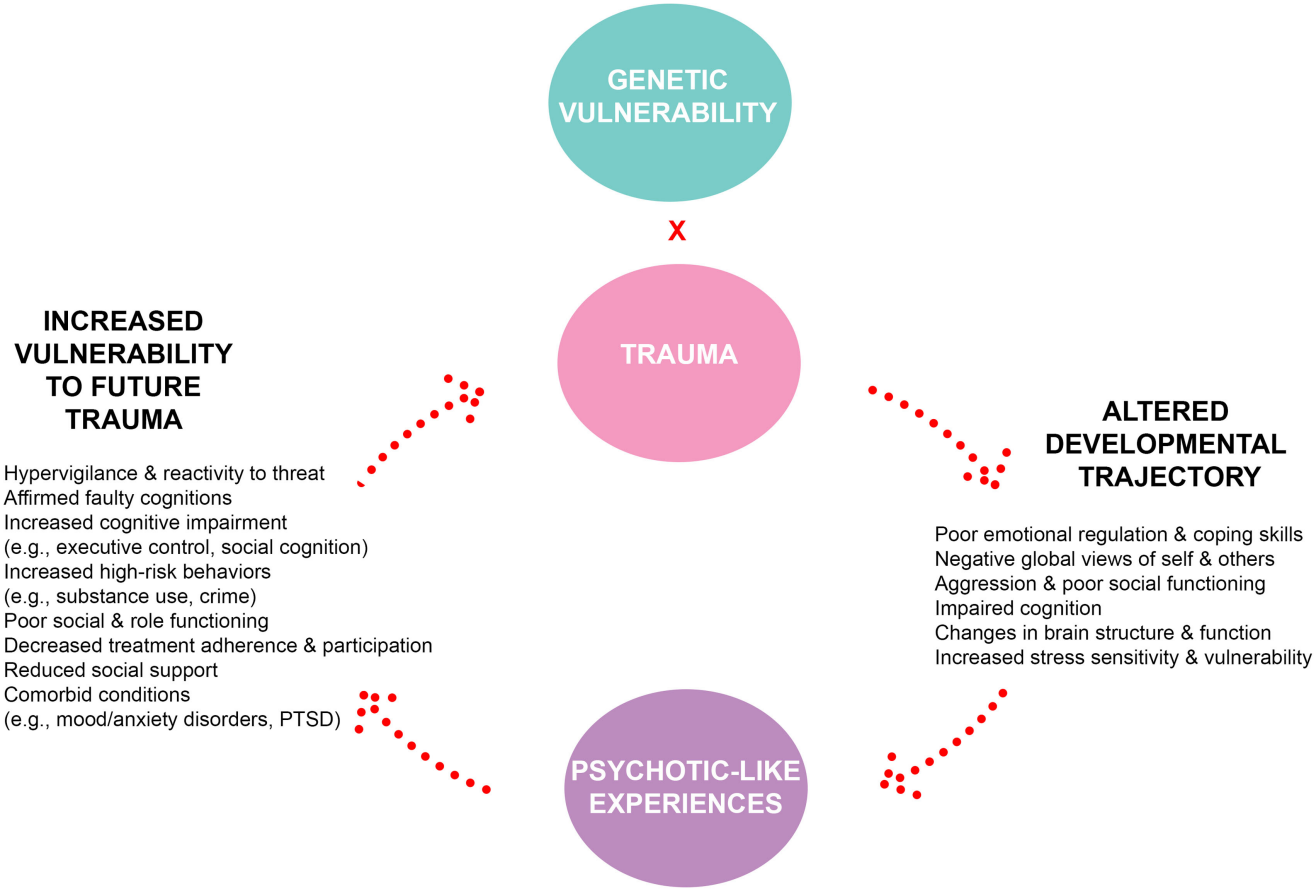
**The cycle of trauma, psychotic-like experiences, and vulnerability**.

Given the high prevalence and relevance of trauma in the CHR syndrome, which we have outlined above, the next step is translating this knowledge to improve interventions for this population. Trauma and psychosis are two of the most difficult clinical symptoms to target and manage in psychiatric treatment; combined, they are considered by most mental health providers to be one of the most complex forms of mental illness, usually requiring a greater level of care. Typically, when individuals present to clinical treatment settings for either symptoms of trauma or psychosis, they are often referred to clinics with an expertise in one of the two areas of concern because few programs provide integrated care for both issues (159). Consequently, there is a growing need for treatment settings that provide clinical expertise on both trauma and psychosis. Given the complex interplay of symptoms, the current model of the trauma-psychosis cycle (see Figure 3) suggests that CHR individuals who present with trauma history do not share the same expected course of treatment and recovery as those without a history of trauma. As shown by Cragin and colleagues (159), there is a growing need to address trauma in EP care. Recent research on trauma treatment in psychosis has shown the impact of prolonged exposure, an evidence-based trauma treatment, on reducing trauma symptoms and psychosis in individuals with chronic schizophrenia and comorbid trauma (160). However, many clinicians are concerned that addressing trauma in treatment may trigger worsening of psychosis (161). Given the lack of attention to trauma-focused treatments in psychosis, it is no surprise that even less has been developed in EP, despite the preponderance of first episode and CHR individuals who endorse CT. To date, no clinical trials have been published evaluating interventions for trauma in CHR youth. Currently, many CHR clinics utilize treatment based on cognitive–behavioral therapy (CBT) methods for individual treatment and family-based treatments [e.g., multi-family group (MFG) or functional family therapy (FFT)] to address social stress and support. The addition of components from Trauma-Focused Cognitive Behavioral Therapy (TF-CBT) represent a potential approach to providing trauma-informed care for CHR individuals that is consistent with prominent treatment models for this population. Both CBTp and TF-CBT start with providing psychoeducation and enhancing coping skills, then introduce methods of cognitive coping, before providing opportunities to address psychotic symptoms, or trauma symptoms *via* exposure. Similar to MFG and FFT, TF-CBT also integrates family members to maintain support for the individual and ensure generalization outside of the therapeutic context. While TF-CBT is a promising approach for the CHR population, it has not yet been fully developed nor evaluated. This is a critical area of future research.

## Implications and Future Directions

Overall, recent findings continue to provide supporting evidence for higher rates of trauma among CHR individuals compared to HC, consistent with Kraan and colleagues (5). Emerging studies continue to show a strong relationship between CT and severity of psychosis symptoms in the CHR population (34, 45, 51). Studies of CHR individuals with CT also repeatedly demonstrate a negative relationship with cognitive, clinical, and social functioning outcomes overtime (34, 59). Trauma appears to predict conversion to psychosis, but may not function independently of other known risk factors such as more severe positive symptoms, cognition, and functioning (16). While CHR individuals may demonstrate heightened stress reactivity at the clinical and biological levels (119, 134), the role of SLEs in triggering transition to psychosis has not been clearly substantiated. As a result, the current proposed model of the trauma-psychosis cycle (Figure 3) focuses solely on CT and not SLE. However, SLE may be an important area to consider when refining the proposed trauma–psychosis model when additional studies provide a richer understanding of its influence on psychosis risk. Finally, the mechanisms underlying the relationship of trauma to psychosis onset in CHR individuals are not fully understood, although there is some evidence for both cognitive and biological (HPA axis, gene–environment interaction) models (111, 141). Most importantly, no appropriate interventions have been developed and validated specifically for trauma in CHR individuals, despite an enormous need for such an approach. The overall findings suggest that the study of trauma, stress, and psychosis risk is still in its early stages and requires continued work. Several suggestions are provided to further research and clinical interventions in addressing the role of trauma and SLEs in psychosis among CHR individuals.

### Recommendations for Future Research

First, larger sample sizes in CHR trauma studies would support stronger inferences in research findings *via* increased statistical power that allows for testing of interaction models, mechanistic mediation models, and simultaneous testing of multiple predictors of outcomes. Adequate representation of minority groups and more international research would help to evaluate potential demographic differences. Second, inclusion of a psychiatric control group (e.g., mood and anxiety disorders without psychotic-like experiences) would prove useful in delineating what is unique to CHR individuals and what is shared with other symptom domains. Third, a standardized measure, validated for the adolescent/young adult population would help to compare across studies and assess cohorts longitudinally. The measure should assess both the number and age of occurrence of traumatic events in order to investigate whether there is a “critical period” for CT and to test stress-sensitization models.

Moving forward, the research definitions for trauma types and SLEs should be consistent and specific in order to facilitate comparison of research results across studies. For instance, some measures assess narrow definitions of trauma while other “trauma” measures also include events that are less severe and are better categorized as SLE. As another example, there is great disparity between individuals and families concerning the definition of childhood physical abuse. Researchers would benefit by providing participants with an operational definition of childhood physical abuse to help increase their responses’ internal validity. Similarly, differences in the definitions of abuse across cultures should also be investigated to clarify the constructs that are measures as part of a study. In addition, the current studies examined suggest that sexual abuse history is a prominent area that demands greater focus and consideration in CHR population research, given the psychological cost of illness that may follow. Based on the current review’s proposed conceptualization of trauma and psychosis risk occurring in a cyclical and repeated pattern, it is suggested that future studies on CHR individuals should consider examining the influence of complex trauma (i.e., multiple types of trauma) on psychosis risk. Additional variables to consider would be the severity and duration of trauma experiences as well as differentiating between a single traumatic event and chronic abuse, with the latter potentially conferring greater risk. Furthermore, research should delve deeper into gender differences among CHR individuals with trauma, given evidence of differential rates and effects in psychosis (42, 53).

### Recommendations for Treatment and Interventions

For clinical recommendations and improved delivery of service, it would be important for clinicians to determine a treatment plan that considers both trauma and psychosis symptoms. A decision-tree process that decides primary areas to initially target would be helpful and should be a focus of future clinical research (90). Clinicians should be able to determine whether trauma is a significant centerpiece of the presenting problem or a complicating factor that aggravates the individual’s psychosis symptoms. Case conceptualizations should also consider modifications of standard treatment when necessary in order to better address the client’s needs. During intake and clinical evaluations, it may be useful to create a timeline of CHR individual’s trauma and SLEs in relation to their other clinical symptoms and associated functional decline. This documented information may prove imperative use for case conceptualization and treatment planning. In reference to the trauma-psychosis cycle (Figure 3), the timeline of events in a CHR individual’s life may give helpful information into the nature of their trauma history, its severity, and the level of treatment required.

The accumulated knowledge on trauma and psychosis thus far highlights that children and youth who experience CT and/or SLEs should be referred for immediate clinical evaluation and intervention. In particular, youth who report early bullying experiences should be taken seriously, as it can be one of the earliest forms of social stress that persists and influences various domains of functioning and well-being. Individuals experiencing psychotic-like symptoms should be encouraged to seek treatment to boost their cognitive and behavioral coping skills in order to help them combat increased vulnerability to future trauma. Families and parents have a pivotal role in increasing the effectiveness of any treatment intervention. As demonstrated by the trauma-psychosis cycle (Figure 3), the experience of trauma can be pervasive and persistent. Clinicians are strongly urged to involve parents, family members, or other significant people in treatment with CHR youth dealing with trauma and psychosis to enhance their social support system and buffer them against additional stressors.

A particular challenge is that many clinicians working with adolescents have not received sufficient training regarding psychotic-spectrum conditions, and specialists in EP may not have sufficient training in trauma treatment (159). Broad availability of training across clinical degree programs in the US regarding assessment and treatment of psychosis would help to improve community providers’ accurate detection of potential CHR syndromes in traumatized youth. Further, training for coordinated specialty care programs that treat EP should include training modules on the appropriate assessment and treatment of trauma.

As advocated by previous research (61, 142), a standard protocol for CT or SLE assessment during all initial patient evaluations should be used in pediatric and behavioral health settings. Due to the sensitive nature of the assessment questions, clinicians and other medical providers should recognize the appropriate format of assessing trauma history in youth (i.e., separately or with their parents/caregivers in the room). Akin to training on suicide risk screening, clinical staff should be knowledgeable on how to identify and assess for trauma when working with CHR youth.

Most importantly, based on the current collective knowledge on trauma and stress in EP, we conclude that evidence-based treatments addressing trauma symptomology in the CHR population is desperately needed. Without a targeted and evidence-based treatment for a large number of CHR youth with trauma history and/or SLE, current interventions may not always be successful in impacting their illness trajectory. Yet, the preliminary outcomes from current studies show promising evidence; with improved understanding of the mechanisms that perpetuate the cycle of trauma among CHR individuals, we can promote resilience and mitigate the vulnerability of CHR individuals to developing a psychotic disorder and improve their chances of recovery from the CHR syndrome.

## Author Contributions

All the authors participated in the writing of the manuscript. DM was the lead author and primary writer. SC and AY contributed in the writing of Section “Mechanisms of Trauma and Stress in the CHR Population.” LK conducted a wide literature review of CHR studies and contributed in the writing of Section “Trauma Experiences in the CHR Population.” SY coauthored Section “Trauma Experiences in the CHR Population” and Section “Recommendations for Future Research.” BS coauthored the “Differential Diagnosis of PTSD and CHR Status” subsection. TN coauthored Sections “Trauma Assessment in the CHR Syndrome” and “Implications and Future Directions” and provided overall feedback on the manuscript. RL was the senior author of the manuscript and provided overall guidance and feedback on topic of trauma, stress, and psychosis risk in CHR group. All authors approved the final version of the manuscript.

## Conflict of Interest Statement

The authors declare that the research was conducted in the absence of any commercial or financial relationships that could be construed as a potential conflict of interest.
